# A feasibility study on using smartphones to conduct short-version verbal autopsies in rural China

**DOI:** 10.1186/s12963-016-0100-6

**Published:** 2016-08-20

**Authors:** Jing Zhang, Rohina Joshi, Jixin Sun, Samantha R. Rosenthal, Miao Tong, Cong Li, Rasika Rampatige, Meghan Mooney, Alan Lopez, Lijing L. Yan

**Affiliations:** 1 The George Institute for Global Health at Peking University Health Science Center, Beijing, People’s Republic of China; 2 The George Institute for Global Health, University of Sydney, Sydney, Australia; 3 The Center for Disease Prevention and Control, Shijiazhuang, Hebei People’s Republic of China; 4 Department of Epidemiology, Brown University School of Public Health, Providence, USA; 5 Institute of Medical Humanities, Peking University Health Science Center, Beijing, People’s Republic of China; 6 The School of Public Health, Peking University Health Science Center, Beijing, People’s Republic of China; 7 Melbourne School of Population and Global Health, University of Melbourne, Melbourne, Australia; 8 Institute for Health Metrics and Evaluation at the University of Washington, Seattle, USA; 9 Duke Global Health Institute, Duke University, Durham, NC USA; 10 Global Health Research Center, Duke Kunshan University, Kunshan, People’s Republic of China

**Keywords:** Smartphone-based, Verbal autopsy, msVA, Cause of death, Township health care provider, Village doctor, Feasibility study

## Abstract

**Background:**

Currently there are two main sources of mortality data with cause of death assignments in China. Both sources–the Ministry of Health-Vital Registration system and the Chinese Disease Surveillance Point system–present their own challenges. A new approach to cause of death assignment is a smartphone-based shortened version of a verbal autopsy survey. This study evaluates the feasibility and acceptability of this new method conducted by township health care providers (THP) and village doctors (VD) in rural China, where a large proportion of deaths occur in homes and cause of death data are inaccurate or lacking.

**Methods:**

The Population Health Metrics Research Consortium mobile phone-based shortened verbal autopsy questionnaire was made available on an Android system-based application, and cause of death was derived using the Tariff method (Tariff 2.0); we called this set of tools “msVA.” msVA was administered to relatives of the deceased by six THPs and six VDs in 24 villages located in six townships of Luquan County, Hebei Province, China. Subsequently, interviews were conducted among 12 interviewers, 12 randomly selected respondents, and five study staff to assess the feasibility and acceptability of using msVA for mortality data collection.

**Results:**

Between July 2013 and August 2013, 268 deaths took place in the study villages. Among the 268 deaths, 227 VAs were completed (nine refusals, 31 migrations and one loss of data due to breakdown of the smartphone). The average time for a VA interview was 21.5 ± 3.4 min (20.1 ± 3.5 min for THP and 23.2 ± 4.1 min for VD). Both THPs and VDs could be successful interviewers; the latter needed more training but had more willingness to implement msVA in the future. The interviews revealed that both interviewers and relatives of the deceased found msVA to be feasible, acceptable, and more desirable than traditional methods. The cost of conducting a new VA was $8.87 per death.

**Conclusions:**

Conduction of msVA by VDs in their own villages was feasible and acceptable in rural northern China. Broader implementation of msVA across rural China could potentially improve the coverage and quality of cause of death data, allowing for better national health evaluation and program planning.

## Background

Data on causes of death are essential for health policy planning and monitoring of health [1]. In rural and remote China, where approximately 650 million people reside, 80 % of all deaths occur at home [2], and fewer than 20 % of cause of death (COD) assignments are completed by certified health professionals; the remaining deaths are reported by unqualified personnel or go unreported altogether [2]. A useful alternative to traditional autopsy in these settings is Verbal Autopsy (VA). VA is an epidemiological tool which is used to determine cause of death via interviewing family members and/or caregivers of the deceased in regions where certified health professionals are limited [3].

Currently, there are two main sources of mortality data in China: the Ministry of Health-Vital Registration (MOH-VR) system and the Sample-based Chinese Disease Surveillance Point (DSP) system. The MOH-VR has paper-based data collection efforts in 41 cities and 85 counties; it is administered to half of the population, including all those in the eastern region, 40 % in the central area, and 10 % in the western regions [4]. The MOH-VR system presents many challenges, such as poorer data quality in new reporting areas, lacking standards for data cleaning, excluding birth registration information, and little coverage in rural regions of China [4]. Due to these limitations, the government of China introduced the Sample-based DSP system, first proposed in 1978, which now reaches approximately 73 million people (5.3 %) in China [5]. The DSP uses paper-based VA questionnaires, which are then analyzed by village doctors (VDs). Though the DSP is an improvement on the MOH-VR, coverage is still limited in rural settings and paper-based data collection is expensive, time-consuming, resource intensive, and requires additional quality control [6].

The Chinese rural health care system is a three-level network comprised of county hospitals, township health care centers and village clinics. The township health care providers (THP) from township health care centers have national medical qualification certificates, whereas doctors from village clinics, often called “village doctors” are not qualified physicians, with limited medical training, basic equipment, and a restricted pharmacopeia [7, 8], so causes of death inferred by them maybe inaccurate.

In the past two decades, several methodological improvements have occurred in data collection and analysis for cause of death assignment. Among them, physician-certified VAs (PCVA) are the most widely used. However, this method relies solely on the expert judgement of physicians based on reported signs and symptoms. Despite the expertise of physicians, this approach is subjective, expensive, and time-consuming [9]. More objective approaches have recently been developed and implemented for the automated assignment of cause of death. It is with the development of automated approaches that mobile phone-based VA could be attempted for the first time. One example approach is InterVA, developed by Umea University [10], which uses a Bayesian probabilistic model informed by a priori approximations of probabilities relating to diseases and symptoms. This method tends to work as well as the a priori approximations that inform the model. Arguably, the most objective automated approach is the Tariff Method developed by the Institute of Health Metrics and Evaluation [11]. The Tariff Method is a simple additive algorithm based on a score, or tariff, for each question item-COD pair that performs as well or better than other analytic methods when validated against the “gold standard” for which the cause of death has been reliably established [11], developed as part of the Population Health Metrics Research Consortium (PHMRC). The latest automated analytic tool of causes of death, called “IHME-SmartVA,” used Tariff 2.0 Method for computer certification of verbal autopsies which performs significantly better than the last version (Tariff 1.0) [12]. An Android-based shortened VA questionnaire is a standardized VA questionnaire developed by the PHMRC, representing a decrease in the total number of questions of 40–55 % from the full PHMRC questionnaire, while the full questionnaire is based on World Health Organization (WHO) standards. The shortened one, although reducing almost 50 % of the questions, has been validated to not have significant declines in accuracy, when compared with the full VA instrument [13]. For this investigation, we focus on the Tariff Method.

Although the Tariff Method has been shown to perform as well as or better than other automated methods [14], it has not been determined whether smartphone data collection is a feasible and acceptable approach by community health workers in rural settings. It is for this reason we aimed to provide empirical evidence regarding the feasibility and acceptability of mobile phone-based shortened VA (msVA) in rural, resource-limited China. The specific objectives of this feasibility study include: a) assessing the feasibility of using msVA in rural, resource-limited China, b) assessing the acceptability of msVA by THPs, VDs, and caregiver respondents, and c) assessing the cost of using msVA in rural China.

## Methods

In order to assess the feasibility, acceptability, and cost of msVA in rural China, first a pilot study was conducted using both THPs and VDs as interviewers to carry out the smartphone-based short-version VA. Second, qualitative interviews were conducted among study personnel to evaluate the acceptability of the new msVA method. Acceptability was assessed by study personnel opinions about the ease of use of msVA, respondent opinions about ease of use, and willingness of both study personnel and respondents to use msVA technology in the future. Finally, the cost of using msVA for cause of death assignment was calculated as projected cost per death in these rural settings.

### msVA pilot study

#### Site selection and study population

Luquan County, Hebei province was selected as the study site. This site was chosen because of previous long-term collaborations. Six out of 12 townships in Luquan County were included in this pilot study. These townships include 24 villages with a total population of 44,761 people. All deaths having occurred between July and August 2013 were selected for msVA data collection administered from December 2013 to January 2014. The respondent(s) invited to participate in the study were the principal person who cared for and was with the deceased during their illness; in the event the deceased was not ill, a close relative of the deceased who was at least 18 years of age was recruited. To be sensitive to grieving, we did not approach relatives for msVA data collection until the deceased had been dead for 5 to 6 months. As compensation, every respondent received a bag of laundry detergent after VA survey completion.

#### msVA interviewers

With the assistance of three project coordinators, six THPs were selected—one from each township health care center, and six VDs were selected—one from each village, with one village being randomly selected from each of the six townships, to conduct the smartphone-based VA. THPs and VDs were recruited by sex, age, education experience, work experience, and performance in daily work to be representative of the population served by each health care center or clinic. Prior to data collection, all THPs and VDs were trained on the msVA method and approved as qualified interviewers. VDs were assigned to conduct data collection in their own villages and the neighboring one to two villages, while the remaining one to two villages were assigned to the THPs.

#### The shortened msVA application

**Fig. 1:**
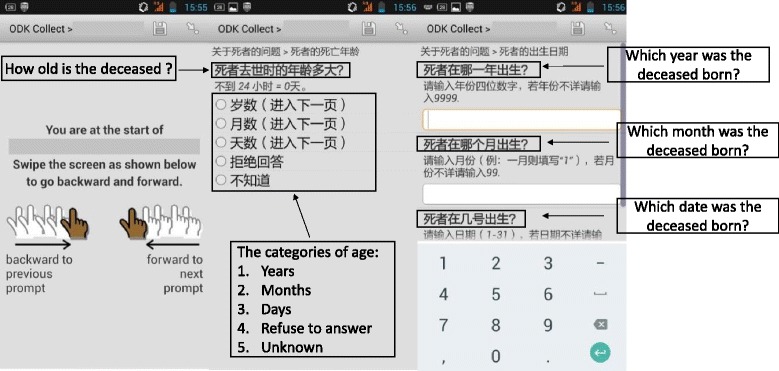
Screen shots of msVA questionnaire

The instrument used was a validated, shortened VA questionnaire, which was reduced by about two-fifths from the full questionnaire without any significant loss of performance compared to the original version [13]. All questionnaires were translated into Chinese with local adaptation. The questionnaire included three main parts: general information, an adult module, and a child/neonate module, which consisted of 109 questions for adults, 69 questions for children, and 67 questions for neonate deaths [13]. Despite the number of questions, the built-in complexity of skip patterns in the shortened questionnaire determines the type and number of questions pertaining to each cause of death, which ultimately contributes to the mean duration of VA interviews. Some photos, sounds (like snoring, wheezing etc.), and other multi-media information were integrated into the application to allow for increased understanding by interviewers and respondents. Some screen shots are shown in Fig. 1.

The msVA was developed for the Android-based free Open Data Kit (ODK) collection tool [15]. Every question was quality-assessed automatically by this system, including value range restrictions, date checking, and logical skipping. The application also has the capability of taking photos of respondents. Also, once data collection is completed, the ODK system prohibits modification of the data.

#### Data upload, transfer, and analysis

**Fig. 2:**
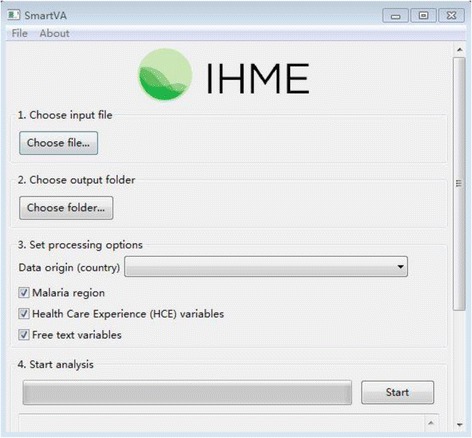
The screen shot of IHME software

Once each interview was complete, the interviewers compiled the completed questionnaires, merged them into one file, and sent them to the county project coordinator weekly. The file was encoded to ensure data confidentiality and security. The George Institute China was responsible for integrating, managing, and transferring the data into a special type (.CSV) via a software, named “ODK Briefcase v1.4 Production” and then importing the files to a software, “IHME-SmartVA,” by which using the Tariff method [11], the causes of death were assigned according to the PHMRC cause list [16]. The screen shot of software is shown in Fig. 2.

#### Quality control of the VA process

The 12 interviewers voice-recorded each interview after receiving permission from the respondent. The recordings were reviewed by four independent research staff for quality control to explore feasibility. Specifically, they assessed: 1) whether the interviewers followed the msVA manual of operation while conducting the VA, 2) how much time was spent for each interview, 3) whether the interviewers understood the questions, and 4) whether the respondents understood the questions. We would retrain the interviewers without good performance on the process of VA survey until eligible.

### Qualitative evaluation

#### Participants

The participants represented the four different roles in the implementation of msVA, including research staff for quality control, project coordinators, VA interviewers and respondents. Our sampling frame was as follows:

1. Four research staff for quality control;
2. One province project coordinator and two county project coordinators who were all familiar with cause of death surveillance;
3. All of the interviewers: six THPs and six VDs;
4. Stratified random sample of respondents. Respondents were selected randomly by village and the cause of death. First, one village in each county was selected at random. Then each of the six villages was randomly assigned a cause of death: one of the principal four diseases in China (cardiovascular disease, stroke, cancer, and diabetes), injury, or neonatal death. Within each village, two respondents were selected at random among those with the assigned cause of death. The total number of respondents was 12.

#### Qualitative interview

Interview guides were developed by research staff from the Peking University for different cadres of study staff and relatives of the deceased. Three focus group interviews were conducted: one for VDs, one for THPs, and one for quality controllers. Focus group interviews for THPs and VDs discussed experiences and attitudes toward msVA. The focus group interviews for quality controllers discussed whether THPs or VDs were suitable for administering msVA. Each focus group lasted about one and a half hours. In addition to focus group interviews, 21 personal in-depth interviews were conducted: three VDs, three THPs, three project coordinators, and 12 respondents. For the first nine personal interviews, these interviews were aimed to assess whether msVA was a feasible and acceptable cause of death surveillance system in China. Each interview lasted about one hour. The stratified random sample of 12 VA respondents had face-to-face interviews at their homes. Respondents were interviewed regarding their willingness to use msVA and their understanding of the msVA questions.

#### Data transcription and analysis

We used thematic framework analysis to analyze the interview transcripts [17], which consists of the following four steps: 1) All recorded materials were transcribed by independent research assistants who understand the dialect of Hebei province, 2) units of meaning were identified and coded from the transcripts, 3) units of meaning were condensed into coded groups, and 4) coded groups were synthesized into descriptions reflecting opinions of msVA of all participants.

### Cost analysis

To project future cost of broader msVA implementation in rural China, we assessed cost per death among the msVA pilot using study cost records. The msVA pilot consisted of five components which required expense. These components include training for interviewers (e.g. materials printing, booking conference room, refreshments, poster for dissemination), personnel salary (e.g. data collection, coordination fee and quality control), infrastructure requirements (e.g. smartphones, communication fee and local travel), and gifts for respondents and miscellaneous (e.g. courier fees–transportation of smartphones, materials, posters).

## Results

Aim 1-Feasibility: Assessing feasibility included whether the interviewers followed the msVA manual of operation while conducting the VA, how much time was spent for each interview, whether the interviewers understood the questions, and whether the respondents understood the questions.

Between July 2013 and August 2013, 268 deaths took place in the study site. Among these 268 deaths, 227 VAs were completed. The remaining 41 (15 %) deaths were excluded due to non-response (nine refusals, 31 migrations and one loss of data due to breakdown of the smartphone). Among the 227 deaths, the top 10 causes of death assigned by msVA were stroke (22.8 %), acute myocardial infarction (11.0 %), asthma/COPD (10.6 %), other cardiovascular disease (9.2 %), other non-communicable diseases (6.4 %), diabetes (5.1 %), falls (4.2 %), lung cancer (3.8 %), prostate cancer (3.6 %), and road traffic (2.7 %).

During all 227 interviews, smartphones and applications worked fairly well, and there were no technical errors in data capturing, data transferring, or data analysis. There were no data lost or missing in the whole process.

### The time used for msVA interviews

Depicted from the voice recordings, the average time cost of an msVA interview was 20.1 ± 3.5 min for the THPs and 23.2 ± 4.1 min for the VDs (21.5 ± 3.4 min among 12 interviewers). THPs spent significantly less time on interviews than VDs (t-test; *P* < 0.05). The details are shown in Table 1.

**Table 1:** Time spent (in minutes) for msVA interviews

Interviewer	Number of respondents	Average time*	Max	Min
Township health care providers	115	20.1 ± 3.5	29	10
Village doctors	112	23.2 ± 4.1	34	11
Total	227	21.5 ± 3.4	-	-

* *P* < 0.05

### The performance of interviewers in msVA pilot

According to quality controller reports, the THPs tended to have better performance administering msVA than the VDs. Three out of six VDs could not completely understand the questions, and communication with respondents was not smooth. However, VDs performance improved after being retrained at least two times.

*They (VDs) stammered in the first two or three interviews early on, and didn’t completely understand all the questions until I retrained them two or three times, while most of the township doctors showed good performance. But for future deployment, all interviewers can master the VA through continuous training exercises.* – Quality controller, Female, 50 years old

Despite the initial challenges faced by VDs, respondents seemed to understand the questions in the interview. One village respondent (Female, 43 years old) in particular noted, “*I think all questions were easy for me, and I did not spend a long time completing the survey.*” A quality controller (Male, 26 years old) also confirmed this point of view, stating, “*From the voice recorders, I can hear clearly that almost every respondent could easily understand what the THPs and VDs said.*”

Aim 2-Acceptability: Acceptability of msVA by township health care providers, VDs, and caregiver respondents was assessed based on reports about the ease of use of the msVA interface and discussion about the msVA protocol compared to the DSP.

### Practicability and operability of smartphones

Most THPs and VDs mentioned the convenience of the smartphone-based VA because of its user-friendly interface. All THPs and the three younger VDs had no trouble uploading the data by themselves. However, the other three older VDs (aged 40+ years) needed additional assistance.

*I can smoothly operate the smartphone so far, although I am getting older. I can use the smartphone to interview because it is easy and user-friendly to operate, and I just follow the procedure, but uploading data using a computer is a little difficult for me. I asked my daughter to help me.* – VD, Male, 52 years old

### Acceptability of msVA by Interviewers

Due to their familiarity with villagers and the village environment, VDs were most accepting of msVA. In comparison, THPs were less accepting, because they had limited contact with villagers and they also usually lived in townships, from which some villages were generally far away. Generally, they needed assistance from the VDs to find the deceased’s homes. All VDs found that scheduling an appointment with caregivers or relatives of the deceased was easy. For example, a VD (Male, 45 years old) said, “*It’s easy to make an appointment with respondents. We know them very well.*” Yet, a THP (Female, 36 years old) felt that, “*It’s not a reasonable long-term plan for us to go to the villages to do the VA interviews. It takes me a long time to travel to the villages, so it is incredibly time consuming.*” After reviewing all transcripts and observing the whole process of the pilot, a project coordinator (Male, 28 years old) noted that, “*All VDs were very familiar with their own villagers. So they can make an appointment with respondents easily, and the villagers have confidence in them.*”

### Acceptability of msVA by caregivers and/or relatives

Most caregivers or relatives were willing to spend about 20 min participating in the msVA survey and also agreed to record the interview. All questions were well-understood by caregiver respondents. Moreover, once agreeing to the interview, respondents were likely to complete the whole interview. Only nine (3 %) refused to participate in the interview, primarily due to overwhelming grief. However, the vast majority of caregivers and relatives were willing. One respondent (from a certain village) said, “*I guess that this interview is one way the government can take care of us so I am willing to be interviewed at any time.*”

### Acceptability of msVA as compared to DSP

Compared with DSP, most THPs, VDs, and all three highly experienced project coordinators preferred msVA.

*Causes of death may not be accurate in the DSP system, but we don’t have any better method to solve this. So why don’t we try the new method? msVA provided the questionnaire as a gold standard, with built-in quality checks, computer-based analytic system for cause of death, and additional quality control using voice recording. I think that’s better for validation of data.* – Project Coordinator, Female, 50 years old.

The VDs expressed a desire to conduct msVA in their own villages in the future, rather than also in other villages—as was done in this study. A VD (Female, 50 years old) said, “*Sometimes I knew causes of death were inaccurate in the DSP, because relatives couldn’t provide the formal certificates of death, and I have not had any solution for this. Now I would like to use this new instrument.*” In terms of workload, another VD (Female, 36 years old) stated, “*I can accept msVA because there are about 20 deaths every year in my village. Though I only need to fill out a form for 10 min using the DSP, it doesn’t put a lot of additional pressure on me to use msVA.*” However, most THPs were not interested in implementing msVA because of their already heavy workload. “*We have a very heavy workload routinely. If they had us do many VA surveys in a short period like this, I would not be willing to join,*” said a THP (Female, 36 years old).

### Aim 3-Average Cost per VA-based death

Costs of the msVA pilot included training sessions, personnel salary, travel for project implementation, data collection, quality control, infrastructure, and some gifts for respondent compensation. Many of these costs, however, are already covered by the governments’ routine funding; therefore new costs needed for future implementation of msVA would only be a portion of the overall cost. The new cost per death of using msVA for cause of death data collection in rural, resource-limited China would be $8.87 US dollars or 55.06 RMB. Both the overall cost per death and new cost per death can be seen in Table 2.

**Table 2:** The cost of establishing a msVA mortality surveillance

Process	Overall cost per death		New cost per death^a^	
	RMB	USD	RMB	USD
Training				
(1) Materials	2.19	0.35	2.19	0.35
(2) Conference room	1.32	0.21	1.32	0.21
(3) Refreshment	0.88	0.14	0.88	0.14
(4) Poster for dissemination	1.52	0.24	1.52	0.24
Personnel				
(1) Data collection	221.93	35.80	-	-
(2) Coordination fee	35.09	5.66	-	-
(3) Quality control	17.54	2.83	17.54	2.83
Infrastructure				
(1) Smartphone	137.12	22.12	-	-
(2) Communication fee	10.53	1.70	10.53	1.70
(3) Local travel	13.16	2.12	-	-
Compensation for respondents				
(1) Gifts	20.00	3.23	20.00	3.23
Miscellaneous				
(1) Courier	1.08	0.17	1.08	0.17
Total	462.36	74.57	55.06	8.87
Exchange rate (1 USD = 6.2 RMB)				

^a^New Costs refer to additional costs required to implement msVA, while overall costs include those already provided by routine government funding

## Discussion

In Luquan County, Hebei Province, a total of 227 VAs were completed in 24 villages. The cost of conducting msVA was $8.87 per death. The average time for a VA interview was 21.5 ± 3.4 min (20.1 ± 3.5 min for THPs and 23.2 ± 4.1 min for VDs). The top 10 causes of death had been ascertained by software “SmartVA,” although these results were not a little different from the top ten figures of Global Burden of Disease in China in 2013 [18], but this is not the study’s concern. More importantly, the msVA illustrated that it could be operated well in rural settings of China. Both THPs and VDs could be successful interviewers; the latter needed more training but had more willingness to implement msVA in the future. The interviews revealed that both interviewers and relatives of the deceased found msVA to be feasible, acceptable, and more desirable than traditional methods.

msVA offers benefits that other cause of death data collection systems lack, such as automated quality assessment and prohibiting data modification after collection. Time analysis and qualitative interviews suggest that msVA is user-friendly and requires minimal additional effort from interviewers. All msVA data can be stored safely on the smartphone and uploaded to a computer secured by password. In addition, the use of smartphones to collect data eliminates costs for printing, posting, sorting and storing of paper forms involved in alternative data collection methods (e.g. MOH-VR and DSP). Among all deaths recorded during the study period, only nine (3 %) caregiver respondents refused to participate. To the best of our knowledge, the only other study on the automated VA tool was conducted by Jon Bird et al. in rural South Africa in 2013 using InterVA. They also found that trained fieldworkers could implement automated VA effectively rather than having to hire more skilled and paid VA staff [19].

The major advantages of the PHMRC short version questionnaire as used in msVA over the WHO short-form VA questionnaire is it’s brevity without a significant drop in performance [13]; the PHMRC questionnaires for adults and children typically contain only 40–55 % of the items required for the WHO short form [13] and are thus much more suited for application in routine cause of death surveillance systems where brevity of interview time is probably the most important consideration, given the annual volume of deaths that would likely be subject to VA.

Cost analysis estimated the cost per death using msVA is $8.87, which is less than the cost in India ($13.24) [6], but still higher than the current payment by the National Health and Family Planning Commission ($1.56 per death) in China. If the previously trained THPs and VDs can train other health care providers in the future, the cost of training could be reduced. Alternatively, using internet-based training would be another cost-effective solution, cutting both the travel costs and the courier fees. To introduce msVA across rural China, the government would need to build a national database of cause of death data; this would also require additional cost. Although the cost of msVA is somewhat higher than the current cost of cause of death data collection in China, msVA offers potential benefits as a feasible method of data collection in rural areas where data are currently inaccurate or lacking.

Given these study findings, it should be feasible for THPs and VDs to complete data collection of the cause of death in each village or township. However, compared to THPs, VDs have greater willingness to do the data collection in their own villages. Under the current 3-level network health care system in China, one VD in each village is responsible for the cause of death surveillance, which is reasonable and practical for msVA implementation. However, there are not enough VDs in the villages of Hebei province due to the shortage of working-age people and the low income associated with working at the community health services. Young doctors are not willing to work at village clinics when they can make more money as hospital health care providers. An additional consequence is that the remaining VDs working at the village level health services are relatively older, and may be more challenged by the manipulation of a smartphone.

There are some limitations in this study. First, each village only had one doctor, which limited our choice of interviewers. Therefore the qualitative data from each village reflects the experiences of that single doctor. Second, the VDs and THPs were not able to upload the msVA data and analyze cause of death locally, but rather, sent encrypted data to the county project coordinators weekly and ascertained cause of death using the off-line software, “IHME-SmartVA.” This approach was used because VDs and THPs had limited access to the University of Washington server–forcing data to be housed on the smartphones for a short period. In the future, building a server in China that can be easily accessed would allow for improved data security and immediate assignment of cause of death on the smartphones at the time of the interview. Third, this study was conducted in Hebei province, a relatively poor province of China. However, there are poorer areas of China where these findings may not be generalizable. Middle-income countries like China and India tend to lack the infrastructure in rural areas to accommodate fixed-line internet access, whereas wireless networks would allow access to telecommunications [20]. As of June 2015, China had 594 million mobile netizens, 89 % of whom use mobile phones to access the internet due to the low cost, especially cellphones with an Android system [21]. If a particular impoverished region cannot access the mobile internet, the original VA method (e.g. paper-based) will have to be used; and if VDs do not have smartphones, governments should cover the cost of cellphones for msVA.

## Conclusions

This study illustrates that msVA is feasible to implement in rural, resource-limited China. Interviewers and relatives of the deceased found msVA to be an acceptable method. Successful interviewers could be THPs or VDs. However, VDs had more willingness to implement msVA and were particularly interested in implementing in their own villages in the future. VDs would need to first be trained on the msVA protocol, and those who are older or not familiar with technology may require multiple trainings. Though msVA is more expensive than the DSP, msVA offers additional data quality assessment and, in some areas, may be a feasible way to collect cause of death data. Finally, msVA offers a new opportunity to obtain higher quality data for cause of death in rural, resource-limited areas.

Future work should include direct assessment of cause of death on smartphones, setting up a server in China to improve data connectivity, creating a national database for cause of death data in China, and broad implementation of msVA for cause of death data collection in rural China. msVA should be implemented in villages across rural China where there is a VD available for administration. VDs should also be asked to administer msVA in neighboring villages where there are no VDs. Broader implementation of msVA across rural China is essential to improving the coverage and quality of cause of death data, allowing for better national health evaluation and program planning.

## Abbreviations

DSP, Disease Surveillance Point; MOH-VR, Ministry of Health-Vital Registration system; msVA, mobile phone-based shortened VA; ODK, Open Data Kit; PCVA, physician-coded VAs; PHMRC, Population Health Metrics Research Consortium; THP, township health care provider; VA, verbal autopsy; VD, village doctor; WHO, World Health Organization
